# Blockage of the mevalonate pathway overcomes the apoptotic resistance to MEK inhibitors with suppressing the activation of Akt in cancer cells

**DOI:** 10.18632/oncotarget.24696

**Published:** 2018-04-13

**Authors:** Mahiro Iizuka-Ohashi, Motoki Watanabe, Mamiko Sukeno, Mie Morita, Ngoc Thi Hong Hoang, Takahiro Kuchimaru, Shinae Kizaka-Kondoh, Yoshihiro Sowa, Koichi Sakaguchi, Tetsuya Taguchi, Toshiyuki Sakai

**Affiliations:** ^1^ Department of Molecular-Targeting Cancer Prevention, Kyoto Prefectural University of Medicine, Kyoto, Japan; ^2^ Division of Endocrine and Breast Surgery, Kyoto Prefectural University of Medicine, Kyoto, Japan; ^3^ Department of Life Science and Technology, School of Life Science and Technology, Tokyo Institute of Technology, Yokohama, Japan

**Keywords:** MEK inhibitor resistance, mevalonate pathway, statin, Akt, apoptosis

## Abstract

With increasing clinical demands for MEK inhibitors in cancer treatment, overcoming the resistance to MEK inhibitors is an urgent problem to be solved. Numerous reports have shown that MEK inhibition results in the activation of PI3K-Akt signaling, which may confer apoptotic resistance to MEK inhibitors. We here demonstrate that the blockade of the mevalonate pathway using the antilipidemic drug statins represses Akt activation following MEK inhibition and induces significant apoptosis when co-treated with CH5126766 or trametinib. These events were clearly negated by the addition of mevalonate or geranylgeranyl pyrophosphate, indicating that the protein geranylgeranylation is implicated in the apoptotic resistance to MEK inhibitors. Furthermore, mechanistically, the combined treatment of CH5126766 with statins upregulated TNF-related apoptosis-inducing ligand (TRAIL), which was dependent on inhibition of the mevalonate pathway and is involved in apoptosis induction in human breast cancer MDA-MB-231 cells. The present study not only revealed that the mevalonate pathway could be targetable to enhance the efficacy of MEK inhibitors, but also proposes that combinatorial treatment of MEK inhibitors with statins may be a promising therapeutic strategy to sensitize cancer cells to apoptosis.

## INTRODUCTION

MEK inhibitors are some of the most successful molecular-targeting drugs in recent years, as symbolized by the approval of trametinib against *BRAF*-mutated melanoma. The developments of novel MEK inhibitors have increased to widely expand their indication for other malignancies. For instance, CH5126766, which is under phase 1 trials, has been expected to be used for *RAS*-mutated cancers with the potential of dual RAF and MEK inhibition [1–3]. However, there has always been the underlying problem of resistance to MEK inhibitors. A number of mechanisms of MEK inhibitor resistance have been reported to date at both of genomic and non-genomic levels. For example, the active mutation of *PIK3CA* gene or loss of function of *PTEN* gene led to the constitutive activation of PI3K-Akt signaling, which is known to confer resistance to MEK inhibition [4–6]. Although *PIK3CA*, *Akt* or *PTEN* genes are intact, MEK inhibition resulted in non-genomic Akt activation through feedback loops mediated by receptor tyrosine kinases, such as EGFR [7–10], FGFR [11, 12], IGF [13, 14], ERBB [12, 15, 16], MET [9] and Axl [17], which may also cause the apoptotic resistance to MEK inhibitors. Thus, the repression of activated PI3K-Akt signaling could be rational to enhance the efficacy of MEK inhibitors, and PI3K inhibitors were expected to be used with MEK inhibitors as combinatorial therapeutics. Indeed, a number of early-phase clinical studies testing the efficacies of the combination of MEK and PI3K inhibition have been performed; however, the results of these studies remain unsatisfactory [18]. Therefore, feasible combination therapy with MEK inhibitors is required to inhibit PI3K-Akt signaling.

The mevalonate pathway, which is an essential metabolic pathway for the biosynthesis of cholesterol, has been heavily investigated as one of the most important metabolisms related to cancer. Indeed, the oral administration of statins, which inhibit the rate-limiting enzyme in the mevalonate pathway HMG-CoA reductase, has been reported to improve the prognosis of breast cancer [19, 20], prostate cancer [21] and colorectal cancer [22]. Mechanistically, downstream metabolites of the mevalonate pathway, isoprenoids, are crucial for prenylation of small GTPases, such as RAS, Rho and Rac, which are implicated in several malignant features such as proliferation, survival, migration and angiogenesis [23–25]. However, little is known as to whether the mevalonate pathway is involved in the resistance to molecular-targeting drugs such as MEK inhibitors.

In the present study, we examined whether the blockade of the mevalonate pathway affected the sensitivity to MEK inhibitors in cancer cells. We here show that statins treatment suppressed Akt activation induced by MEK inhibitor treatment and overcame the apoptotic resistance to CH5126766 and trametinib dependently on the inhibition of the mevalonate pathway, particularly protein geranylgeranylation. Our results suggest that the metabolic pathways, such as the mevalonate pathway, can be therapeutically targeted to increase the efficacy of MEK inhibition, and also indicate that statins can be a feasible avenue for combinatorial treatment with MEK inhibitors.

## RESULTS

### Statins enhance the sensitivity to MEK inhibitors

**Figure 1:**
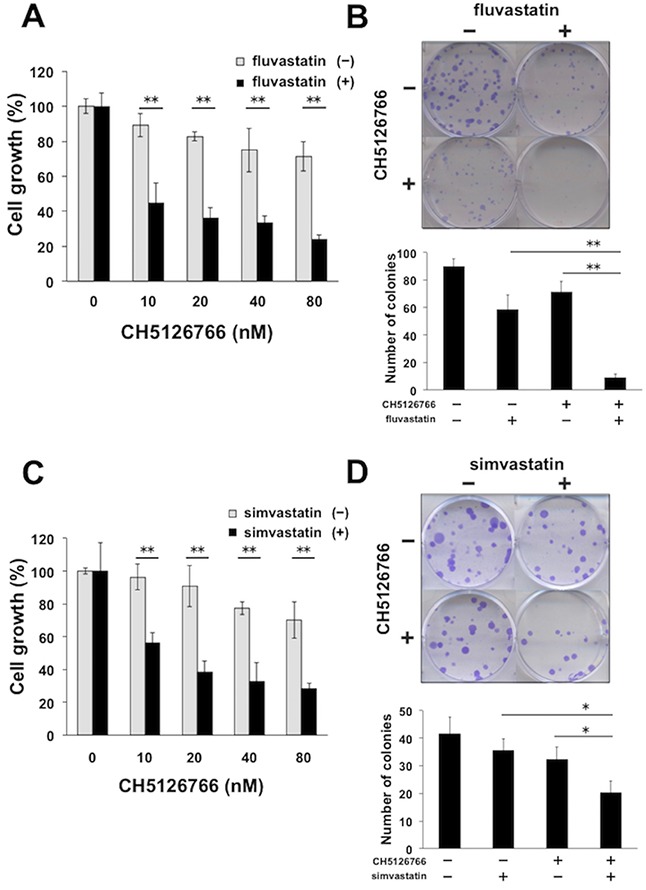
Statins enhance the sensitivity to MEK inhibitors **(A)** Growth inhibitory effects of CH5126766 with or without fluvastatin on MDA-MB-231 cells. Cells were treated with CH5126766 at the indicated concentrations with or without fluvastatin (0.3 μM) for 72 h, and the cell viability was measured with a Cell Counting Kit-8 assay. Data obtained with DMSO with or without fluvastatin was taken as 100%. Columns, means of triplicate data; bars, standard deviation (SD); ^**^, P < 0.01. **(B)** Suppression of colony formation by the combined treatment of CH5126766 with fluvastatin. MDA-MB-231 cells were treated with CH5126766 (40 nM) and/or fluvastatin (0.3 μM) for 72 h. After further incubation for colony formation with fresh medium, the number of colonies was counted. (upper panel) The representative images of stained colonies are shown. (lower panel) Colony numbers are shown in the graph. Columns, means of triplicate data; bars, SD; ^**^, P < 0.01. **(C)** Growth inhibitory effects of CH5126766 with or without simvastatin on MDA-MB-231 cells. Cells were treated with CH5126766 at the indicated concentrations with or without simvastatin (0.3 μM) for 72 h, and the cell viability was measured with a Cell Counting Kit-8 assay. Data obtained with DMSO with or without simvastatin was taken as 100%. Columns, means of triplicate data; bars, standard deviation (SD); ^**^, P < 0.01. **(D)** Suppression of colony formation by the combined treatment of CH5126766 with simvastatin. MDA-MB-231 cells were treated with CH5126766 (20 nM) and/or simvastatin (0.3 μM) for 72 h. After further incubation for colony formation with fresh medium, the number of colonies was counted. (upper panel) The representative images of stained colonies are shown. (lower panel) Colony numbers are shown in the graph. Columns, means of triplicate data; bars, SD; ^*^, P < 0.05.

In order to investigate whether the mevalonate pathway affects the sensitivity to MEK inhibitors, we treated human breast cancer MDA-MB-231 cells harboring *KRAS* and *BRAF* mutations with a MEK inhibitor, CH5126766, with or without statins, which inhibits HMG-CoA reductase, the rate-limiting enzyme in the mevalonate pathway. The combined treatment of CH5126766 with fluvastatin demonstrated more significant reduction in cell growth in a dose-dependent manner than the single treatment of CH5126766 (Figure 1A). We also confirmed the marked combined effects of CH5126766 at 40 nM and fluvastatin at 0.3 μM on the suppression of the colony formation of the cells (Figure 1B). We next co-treated cells with CH5126766 and another statin, simvastatin, and similar results were obtained in the suppression of cell growth (Figure 1C) and colony formation (Figure 1D). These results suggest that statins-mediated inhibition of the mevalonate pathway could increase the efficacy of MEK inhibitors.

### The co-treatment of MEK inhibitors with statins induces apoptosis with the suppression of Akt activation

**Figure 2:**
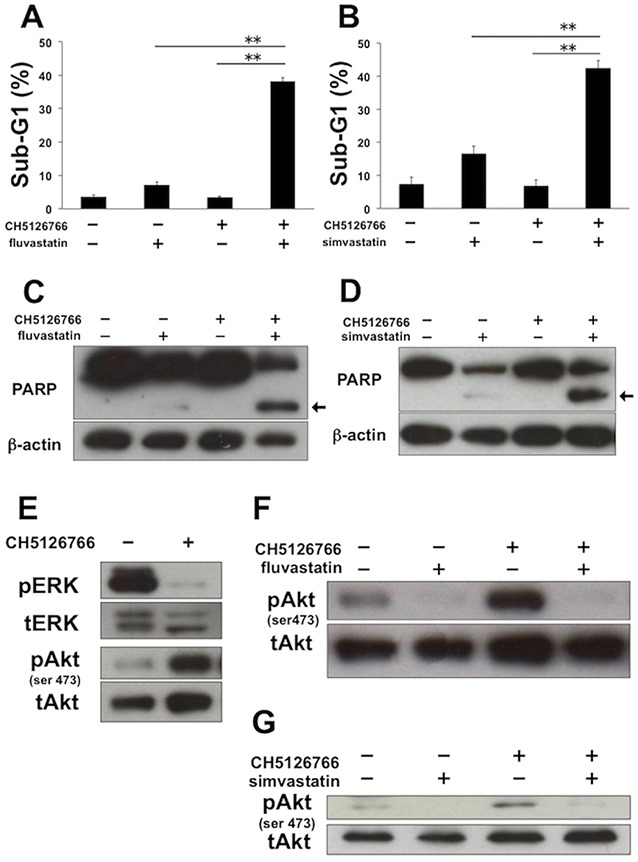
The combined treatment of MEK inhibitor with statins induces apoptosis with the suppression of Akt activation in MDA-MB-231 cells **(A)** The sub-G1 populations after the combined treatment of CH5126766 with fluvastatin. Cells were treated with CH5126766 (40 nM) and/or fluvastatin (0.3 μM) for 72 h. DNA contents of the cells were analyzed by flow cytometer. Columns, means of triplicate data; bars, SD; ^**^, P < 0.01. **(B)** The sub-G1 populations after the combined treatment of CH5126766 with simvastatin. Cells were treated with CH5126766 (20 nM) and/or simvastatin (0.3 μM) for 72 h. DNA contents of the cells were analyzed by flow cytometer. Columns, means of triplicate data; bars, SD; ^**^, P < 0.01. **(C)** The cleavage of PARP after the combined treatment of CH5126766 with fluvastatin. Cells were treated with CH5126766 (40 nM) and/or fluvastatin (0.3 μM) for 48 h, and cleaved PARP was analyzed by Western blotting. The arrow indicates the cleaved form of PARP. β-Actin was used as a loading control. **(D)** The cleavage of PARP after the combined treatment of CH5126766 with simvastatin. Cells were treated with CH5126766 (20 nM) and/or simvastatin (0.3 μM) for 48 h, and cleaved PARP was analyzed by Western blotting. The arrow indicates the cleaved form of PARP. β-Actin was used as a loading control. **(E)** The phosphorylation status of ERK and Akt after the treatment of CH5126766. Cells were treated with CH5126766 (20 nM) for 24 h, and phosphorylated ERK and Akt were analyzed by Western blotting. **(F)** The phosphorylation status of Akt after the combined treatment of CH5126766 with fluvastatin. Cells were treated with CH5126766 (40 nM) and/or fluvastatin (0.3 μM) for 48 h, and phosphorylated Akt was analyzed by Western blotting. **(G)** The phosphorylation status of Akt after the combined treatment of CH5126766 with simvastatin. Cells were treated with CH5126766 (20 nM) and simvastatin (0.3 μM) for 48 h, and phosphorylated Akt was analyzed by Western blotting.

We carried out flow cytometric analysis of the cell cycle and apoptosis to further investigate the mechanisms of the combined effects of MEK inhibitors with statins. Regarding the cell cycle, fluvastatin or simvastatin with or without CH5126766 caused G1 arrest in MDA-MB-231 cells (Supplementary Figure 1A and 1B, respectively). Next we analyzed sub-G1 population of cells for the detection of apoptosis. Although sub-G1 cells dose-dependently increased after the treatment of fluvastatin or simvastatin, each statin at 0.3 μM hardly increased sub-G1 cells (Supplementary Figure 2A). However, the co-treatment of 40 nM CH5126766 with 0.3 μM fluvastatin resulted in a significant increase in the sub-G1 population (Figure 2A), indicating that the combination of both induced apoptosis. The same tendency was observed with the co-treatment of CH5126766 with simvastatin (Figure 2B). To further confirm the induction of apoptosis, we carried out Western blotting to detect the cleavage of PARP. Consistent with the results of flow cytometric analysis, 0.3 μM statins only showed slight cleavages of PARP (Supplementary Figure 2B), while the co-treatment of CH5126766 with fluvastatin (Figure 2C) or simvastatin (Figure 2D) clearly induced the cleavages of PARP. Furthermore, we tested whether another MEK inhibitor, trametinib, also induces apoptosis when combined with statins. As shown in Supplementary Figure 3A and 3B, significant increases in the sub-G1 population were also observed when cells were simultaneously treated with trametinib and fluvastatin or simvastatin. These results suggest that statins treatment could overcome the apoptotic resistance to MEK inhibitors.

As the activation of Akt signaling following inhibition of the MEK pathway is known to cause apoptotic resistance to MEK inhibitors [7–17], we next analyzed the phosphorylation status of Akt by Western blotting after MDA-MB-231 cells were treated with CH5126766. As previously reported, the level of phosphorylated Akt was elevated when ERK was dephosphorylated by CH5126766 treatment for 24 h (Figure 2E). Of particular note is that CH5126766-mediated activation of Akt was suppressed by the addition of fluvastatin (Figure 2F) or simvastatin (Figure 2G).

This scenario raises the possibility that MEK inhibitor-mediated Akt activation could intensify the mevalonate pathway since PI3K-Akt signaling increases the expressions of SREBPs [26, 27] and the activity of SREBP2 [28], which are transcriptional factors of HMG-CoA reductase. We then analyzed the expression levels of HMG-CoA reductase in cells treated with CH5126766 or trametinib. As shown in Supplementary Figure 4, we observed no alteration of the expression of HMG-CoA reductase at any time point, suggesting that MEK inhibitors do not activate the mevalonate pathway.

### TRAIL is involved in the combinatorial apoptosis induced by CH5126766 with fluvastatin in MDA-MB-231 cells

**Figure 3:**
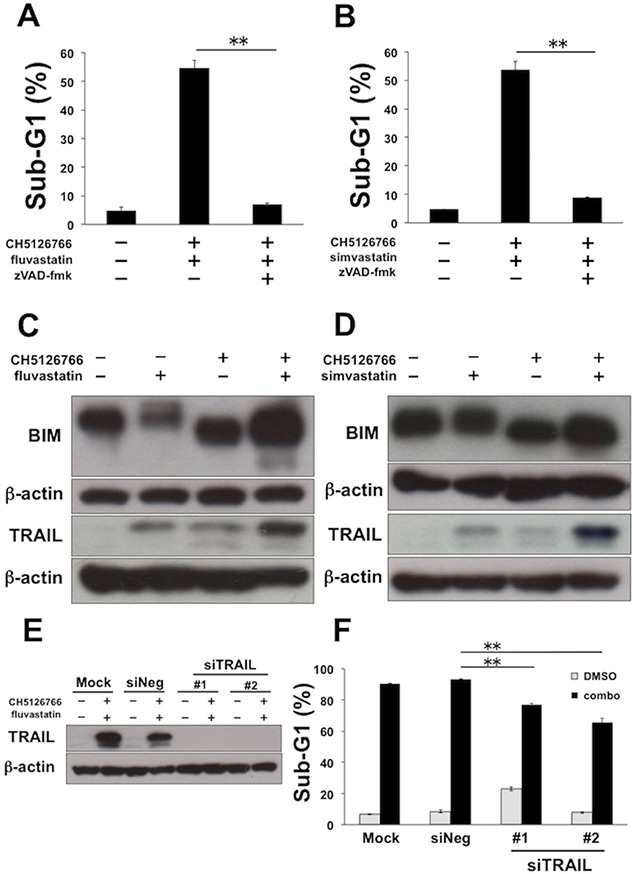
TRAIL is partially required for the apoptosis induced by CH5126766 and fluvastatin in MDA-MB-231 cells **(A)** The sub-G1 populations after the combined treatment of CH5126766 with fluvastatin in the absence or presence of the pan caspase inhibitor zVAD-fmk. Cells were treated with CH5126766 (40 nM) and fluvastatin (0.3 μM) for 72 h with or without zVAD-fmk (20 μM). DNA contents of the cells were analyzed by flow cytometer. Columns, means of triplicate data; bars, SD; ^**^, P < 0.01. **(B)** The sub-G1 populations after the combined treatment of CH5126766 with simvastatin in the absence or presence of the pan caspase inhibitor zVAD-fmk. Cells were treated with CH5126766 (20 nM) and simvastatin (0.3 μM) for 72 h with or without zVAD-fmk (20 μM). DNA contents of the cells were analyzed by flow cytometer. Columns, means of triplicate data; bars, SD; ^**^, P < 0.01. **(C)** The expression of BIM and TRAIL after the combined treatment of CH5126766 with fluvastatin. Cells were treated with CH5126766 (40 nM) and/or fluvastatin (0.3 μM) for 48 h, and the expressions of BIM and TRAIL was analyzed by Western blotting. β-Actin was used as a loading control. **(D)** The expression of BIM and TRAIL after the combined treatment of CH5126766 with simvastatin. Cells were treated with CH5126766 (20 nM) and/or simvastatin (0.3 μM) for 48 h with or without zVAD-fmk (20 μM). DNA contents of the cells were analyzed by flow cytometer. **(E)** The knockdown efficacy of siTRAIL validated by Western blotting. β-Actin was used as a loading control. **(F)** The sub-G1 populations after the combined treatment of CH5126766 with fluvastatin in the TRAIL-depleted cells. Cells were treated with CH5126766 (40 nM) and fluvastatin (0.3 μM) for 72 h after the transfection of each siRNA. DNA contents of the cells were analyzed by flow cytometer. Columns, means of triplicate data; bars, SD; ^**^, P < 0.01.

We further examined more detailed mechanisms of the synergistic apoptosis by the combination of CH5126766 and statins in MDA-MB-231 cells. First, we confirmed that apoptosis induced by the co-treatment with CH5126766 with fluvastatin or simvastatin was significantly negated by the addition of the pan-caspase inhibitor zVAD-fmk (Figure 3A and 3B), indicating that the combinatorial apoptosis was induced in a caspase-dependent manner. Next, we analyzed the apoptotic molecules that were involved in apoptosis. Consistent with the previous reports demonstrating that upregulation of the intrinsic apoptotic protein BIM was crucial to MEK inhibitor-mediated apoptosis in *KRAS* or *BRAF* mutant cancers [29, 30] we observed slight increases of BIM expression in cells co-treated with CH5126766 and statins compared with each single treatment (Figure 3C and 3D). On the other hand, as recent reports showed that extrinsic apoptotic protein TRAIL could overcome the apoptotic resistance to RAF and/or MEK inhibitors [31, 32], we evaluated the expression of TRAIL after the treatment of CH5126766 with or without statins. As shown in Figure 3C and 3D, a significant increase of TRAIL was observed in the combined treatment of CH5126766 with fluvastatin (Figure 3C) or simvastatin (Figure 3D), whereas slight increases were observed with each single treatment. To further confirm the contribution of the upregulation of TRAIL to apoptosis, we performed a knockdown assay of TRAIL using two siRNAs targeting different sequences of the TRAIL gene (Figure 3E). Apoptosis induced by the combined treatment of CH5126766 with fluvastatin was partially reduced in TRAIL-depleted MDA-MB-231 cells (Figure 3F). These results suggest that the upregulation of TRAIL is at least partially involved in apoptosis induced by CH5126766 with fluvastatin.

### Apoptosis induced by CH5126766 with statins is dependent on inhibition of the mevalonate pathway

**Figure 4:**
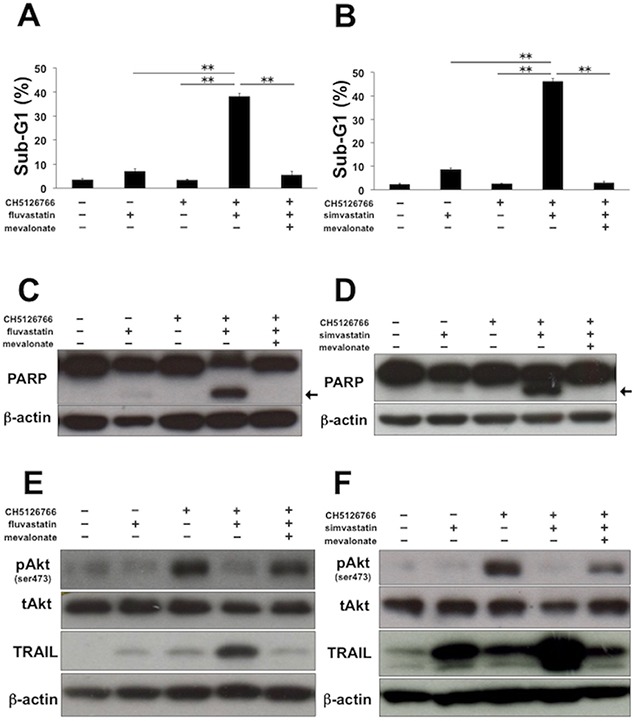
Apoptosis induced by CH5126766 with statins is dependent on inhibition of the mevalonate pathway **(A)** The sub-G1 populations after the combined treatment of CH5126766 with fluvastatin in the absence or presence of mevalonate. MDA-MB-231 cells were treated with CH5126766 (40 nM) and/or fluvastatin (0.3 μM) for 72 h with or without mevalonate (50 μM). DNA contents of the cells were analyzed by flow cytometer. Columns, means of triplicate data; bars, SD; ^**^, P < 0.01. **(B)** The sub-G1 populations after the combined treatment of CH5126766 with simvastatin in the absence or presence of mevalonate. MDA-MB-231 cells were treated with CH5126766 (20 nM) and/or simvastatin (0.3 μM) for 72 h with or without mevalonate (50 μM). DNA contents of the cells were analyzed by flow cytometer. Columns, means of triplicate data; bars, SD; ^**^, P < 0.01. **(C)** The cleavage of PARP after the combined treatment of CH5126766 with fluvastatin in the absence or presence of mevalonate. MDA-MB-231 cells were treated with CH5126766 (40 nM) and/or fluvastatin (0.3 μM) for 48 h with or without mevalonate (50 μM), and cleaved PARP was analyzed by Western blotting. The arrow indicates the cleaved form of PARP. β-Actin was used as a loading control. **(D)** The cleavage of PARP after the combined treatment of CH5126766 with simvastatin in the absence or presence of mevalonate. MDA-MB-231 cells were treated with CH5126766 (20 nM) and/or simvastatin (0.3 μM) for 48 h with or without mevalonate (50 μM), and cleaved PARP was analyzed by Western blotting. The arrow indicates the cleaved form of PARP. β-Actin was used as a loading control. **(E)** The phosphorylation status of Akt and the expression of TRAIL after the combined treatment of CH5126766 with fluvastatin in the absence or presence of mevalonate. MDA-MB-231 cells were treated with CH5126766 (40 nM) and/or fluvastatin (0.3 μM) for 48 h with or without mevalonate (50 μM), and the phosphorylated Akt and the expression of TRAIL were analyzed by Western blotting. β-Actin was used as a loading control. **(F)** The phosphorylation status of Akt and the expression of TRAIL after the combined treatment of CH5126766 with simvastatin in the absence or presence of mevalonate. MDA-MB-231 cells were treated with CH5126766 (20 nM) and/or simvastatin (0.3 μM) for 48 h with or without mevalonate (50 μM), and the phosphorylated Akt and the expression of TRAIL were analyzed by Western blotting. β-Actin was used as a loading control.

In order to assess whether the apoptosis induced by CH5126766 with statins is dependent on the inhibition of the mevalonate pathway, we co-treated MDA-MB-231 cells with CH5126766 and statins supplemented with mevalonate. Sub-G1 cells induced by the co-treatment of CH5126766 with fluvastatin or simvastatin were markedly reduced to the level of the single treatment of CH5126766 by the addition of mevalonate (Figure 4A and 4B). Consistently, the cleavage of PARP induced by the co-treatment was also negated by the addition of mevalonate (Figure 4C and 4D). Furthermore, the supplemented mevalonate led to the reactivation of Akt and negated the upregulation of TRAIL (Figure 4E and 4F). These results of the add-back experiments using mevalonate suggest that the combinatorial apoptosis induced by CH5126766 with statins is dependent on inhibition of the mevalonate pathway.

#### Statins suppress MEK inhibitor-induced Akt activation and lead to the combined apoptosis dependently on the inhibition of geranylgeranylation

**Figure 5:**
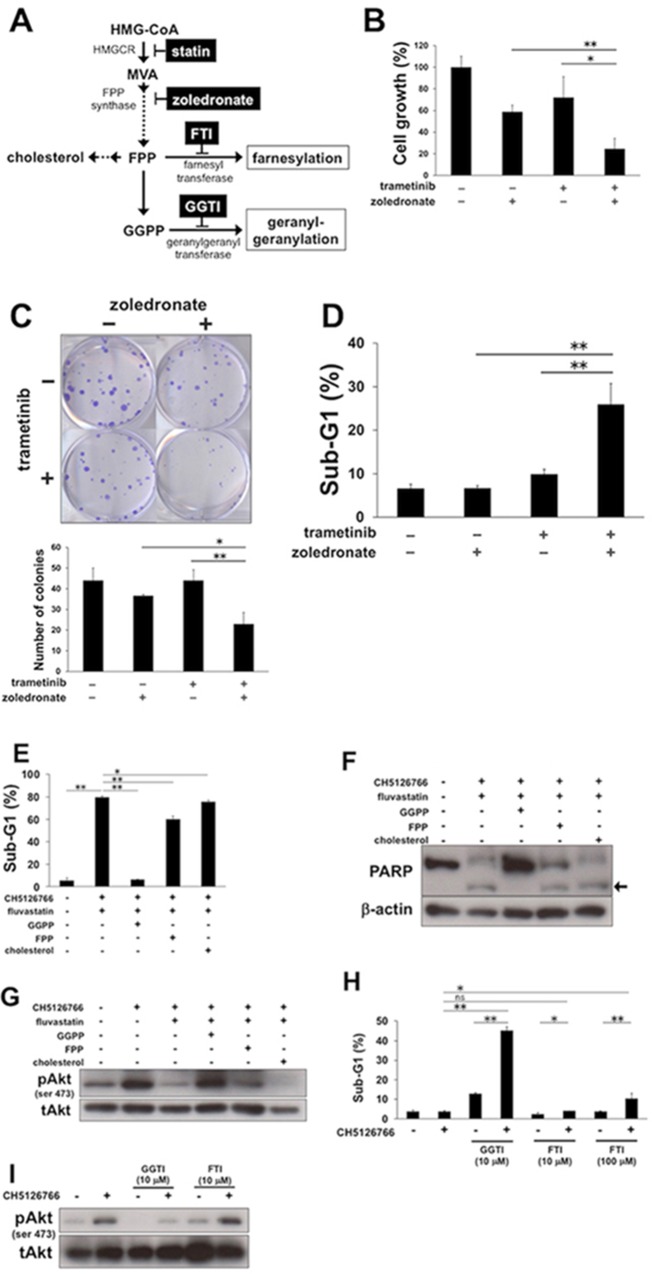
The inhibition of geranylgeranylation is required for statins-mediated suppression of Akt activation and the combined apoptosis **(A)** A simplified schematic of the mevalonate pathway. Dashed arrows indicate that there are multiple steps. HMGCR, HMG-CoA reductase; MVA, mevalonate; FPP, farnesyl pyrophosphate; GGPP, geranylgeranyl pyrophosphate; FTI, farnesyl transferase inhibitor; GGTI, geranylgeranyl transferase inhibitor. **(B)** Growth inhibitory effects of the combined treatment of trametinib with zoledronate on MDA-MB-231 cells. Cells were treated with trametinib (30 nM) and/or zoledronate (30 μM) for 72 h, and the cell viability was measured with a Cell Counting Kit-8 assay. The data obtained with DMSO was taken as 100%. Columns, means of triplicate data; bars, SD; ^**^, P < 0.01; ^*^, P<0.05. **(C)** Suppression of colony formation by the combined treatment of trametinib with zoledronate. MDA-MB-231 cells were treated with trametinib (30 nM) and/or zoledronate (30 μM) for 48 h. After further incubation for colony formation with fresh medium, the number of colonies was counted. (upper panel) The representative images of stained colonies are shown. (lower panel) Colony numbers are shown in the graph. Columns, means of triplicate data; bars, SD; ^**^, P < 0.01; ^*^, P < 0.05. **(D)** The sub-G1 populations after the combined treatment of trametinib with zoledronate. MDA-MB-231 cells were treated with trametinib (30 nM) and/or zoledronate (30 μM) for 48 h. DNA contents of the cells were analyzed by flow cytometer. Columns, means of triplicate data; bars, SD; ^**^, P < 0.01. **(E)** The sub-G1 populations after the combined treatment of CH5126766 with fluvastatin in the absence or presence of GGPP, FPP, or cholesterol. MDA-MB-231 cells were treated with CH5126766 (40 nM) and fluvastatin (0.3 μM) for 72 h with or without GGPP (5 μM), FPP (5 μM), or cholesterol (50 μM). DNA contents of the cells were analyzed by flow cytometer. Columns, means of triplicate data; bars, SD; ^**^, P < 0.01; ^*^, P < 0.05. **(F)** The cleavage of PARP after the combined treatment of CH5126766 with fluvastatin in the absence or presence of GGPP, FPP, or cholesterol. MDA-MB-231 cells were treated with CH5126766 (40 nM) and fluvastatin (0.3 μM) for 48 h with or without GGPP (5 μM), FPP (5 μM), or cholesterol (50 μM). Cleaved PARP was analyzed by Western blotting. The arrow indicates the cleaved form of PARP. β-Actin was used as a loading control. **(G)** The phosphorylation status of Akt after the combined treatment of CH5126766 with or without fluvastatin in the absence or presence of GGPP, FPP, or cholesterol. MDA-MB-231cells were treated with CH5126766 (40 nM) and/or fluvastatin (0.3 μM) for 48 h in the absence or presence of GGPP (5 μM), FPP (5 μM), or cholesterol (50 μM). **(H)** The sub-G1 populations after the combined treatment of CH5126766 with GGTI or FTI. MDA-MB-231 cells were treated with CH5126766 (40 nM) and/or GGTI-298 or FTI-277 at the indicated concentrations for 72 h. DNA contents of the cells were analyzed by flow cytometer. Columns, means of triplicate data; bars, SD; ^**^, P < 0.01; ^*^, P < 0.05; ns, no significant difference. **(I)** The phosphorylation status of Akt after the combined treatment of CH5126766 with/without GGTI or FTI in MDA-MB-231 cells. Cells were treated with CH5126766 (40 nM) in the absence or presence of GGTI-298, or FTI-277 at the indicated concentrations for 48 h, and the phosphorylated Akt was analyzed by Western blotting.

We next investigated which of the mevalonate pathway metabolites, such as farnesyl pyrophosphate (FPP), geranylgeranyl pyrophosphate (GGPP) and cholesterol (Figure 5A), contributed to the combinatory effect of MEK inhibitor with statins. First, we examined whether a nitrogenous bisphosphonate, zoledronate, which inhibits FPP synthase (Figure 5A), showed the combinatorial effect with MEK inhibitor. The co-treatment of trametinib at 30 nM with zoledronate at 30 μM for 72 h significantly suppressed cell growth (Figure 5B) and colony formation of the cells (Figure 5C). Furthermore, this combination expectedly resulted in a significant increase of cells in the sub-G1 population (Figure 5D), confirming that the downstream metabolites of mevalonate confer the apoptotic ability to MEK inhibition. Next, although we observed the reduction in the intracellular cholesterol levels in MDA-MB-231 cells treated with 0.3 μM fluvastatin or simvastatin (Supplementary Figure 5A), the supplementation of cholesterol, which led to a sufficient upregulation of intracellular cholesterol (Supplementary Figure 5B), hardly changed the sub-G1 population induced by the combination of CH5126766 with fluvastatin (Figure 5E). Consistently, the cleaved PARP induced by the combination (Figure 5F) and fluvastatin-mediated suppression of phosphorylated Akt (Figure 5G) did not alter after the cholesterol addition. These results indicate that statins-mediated reduction in intracellular cholesterol levels was not involved in the combined induction of apoptosis. We then considered the possibility that other mevalonate pathway metabolites, FPP or GGPP played a role in this context. While the supplementation of FPP slightly cancelled the combined accumulation of sub-G1 cells (Figure 5E), the supplementation of GGPP almost fully negated the accumulation of sub-G1 cells (Figure 5E) and the cleavage of PARP (Figure 5F) induced by the co-treatment of CH5126766 with fluvastatin. Furthermore, fluvastatin-mediated suppression of Akt activation after CH5126766 treatment was clearly added back by not FPP but GGPP (Figure 5G). Indeed, 0.3 μM fluvastatin and simvastatin increased the accumulation of unprocessed Rap1 (Supplementary Figure 5C), indicating that statins actually inhibited protein geranylgeranylation. To further confirm these observations, we analyzed the sub-G1 population in cells co-treated with CH5126766 and geranylgeranyl transferase inhibitor (GGTI) or farnesyl transferase inhibitor (FTI). We expectedly observed that 10 μM GGTI-298 enhanced the apoptosis induction of CH512676, even though 100 μM FTI-277 with CH512676 showed only slight increase of sub-G1 cells (Figure 5H). Consistently, not FTI-277 but GGTI-298 suppressed the phosphorylation of Akt induced by CH5126766 (Figure 5I), phenocopying the action of statins. Taken together, statins-mediated inhibition of protein geranylgeranylation can be critical to suppress the activation of Akt and overcome apoptotic resistance to MEK inhibition.

### Statins overcome apoptotic resistance to MEK inhibitors in other cancer cell lines

**Figure 6:**
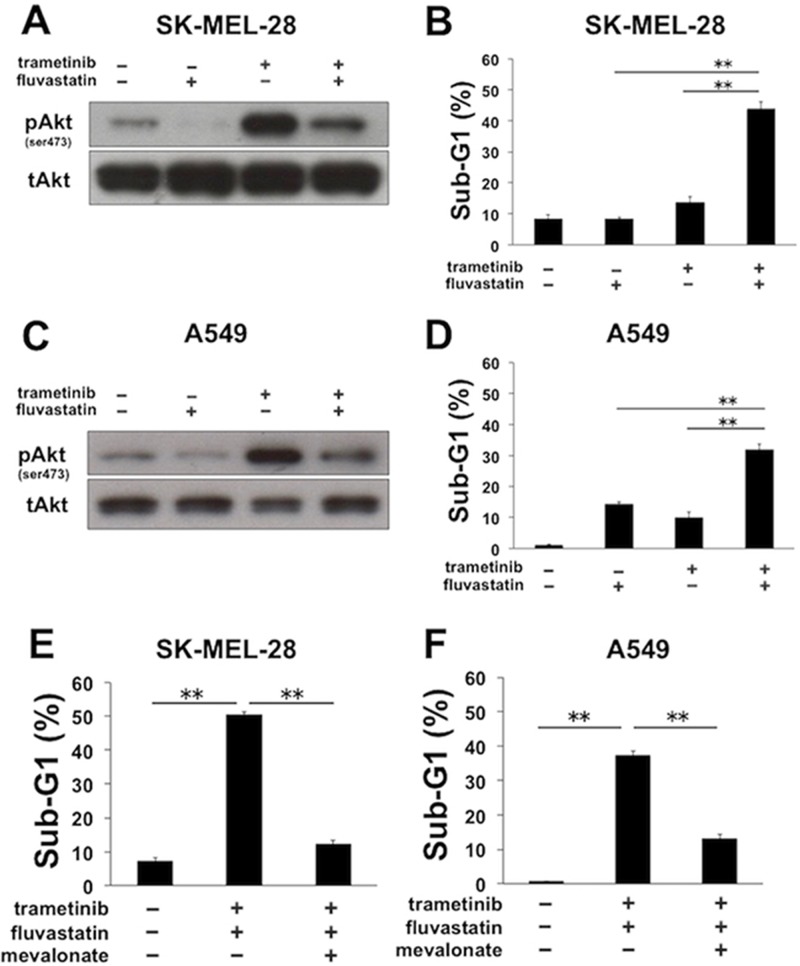
Fluvastatin overcomes apoptotic resistance to MEK inhibitors in a mevalonate pathway-dependent manner in SK-MEL-28 and A549 cells **(A)** The phosphorylation status of Akt after the combined treatment of trametinib with fluvastatin in SK-MEL-28 cells. Cells were treated with trametinib (5 nM) and/or fluvastatin (1 μM) for 48 h, and the phosphorylated Akt was analyzed by Western blotting. **(B)** The sub-G1 populations after the combined treatment of trametinib with fluvastatin in SK-MEL-28 cells. Cells were treated with trametinib (5 nM) and/or fluvastatin (1 μM) for 72 h. DNA contents of the cells were analyzed by flow cytometer. Columns, means of triplicate data; bars, SD; ^**^, P < 0.01 **(C)** The phosphorylation status of Akt after the combined treatment of trametinib with fluvastatin in A549 cells. Cells were treated with trametinib (40 nM) and/or fluvastatin (2 μM) for 48 h, and the phosphorylated Akt was analyzed by Western blotting. **(D)** The sub-G1 populations after the combined treatment of trametinib with fluvastatin in A549 cells. Cells were treated with trametinib (40 nM) and/or fluvastatin (2 μM) for 72 h. DNA contents of the cells were analyzed by flow cytometer. Columns, means of triplicate data; bars, SD; ^**^, P < 0.01 **(E)** The sub-G1 populations after the combined treatment of trametinib with fluvastatin in the absence or presence of mevalonate. SK-MEL-28 cells were treated with trametinib (5 nM) and fluvastatin (1 μM) for 72 h with or without mevalonate (50 μM). DNA contents of the cells were analyzed by flow cytometer. Columns, means of triplicate data; bars, SD; ^**^, P < 0.01 **(F)** The sub-G1 populations after the combined treatment of trametinib with fluvastatin in the absence or presence of mevalonate. A549 cells were treated with trametinib (40 nM) and fluvastatin (2 μM) for 72 h with or without mevalonate (50 μM). DNA contents of the cells were analyzed by flow cytometer. Columns, means of triplicate data; bars, SD; ^**^, P < 0.01.

We examined whether the combinatorial apoptosis induced by the co-treatment of MEK inhibitors with statins occurs in other cancer cell lines. Human melanoma SK-MEL-28 cells with *BRAF* mutation are known to be sensitive to trametinib; however, the level of phosphorylated Akt was clearly elevated after trametinib treatment (Figure 6A). Trametinib-mediated Akt activation was successfully suppressed by the concomitant treatment with fluvastatin in SK-MEL-28 cells (Figure 6A), as similarly observed in MDA-MB-231 cells (Figure 2F and 2G). Furthermore, the significant increase of the sub-G1 population was also observed with combined treatment of trametinib with fluvastatin in SK-MEL-28 cells (Figure 6B). In addition, in human non-small cell lung cancer A549 cells with *KRAS* mutation, trametinib-mediated elevation of phosphorylated Akt was suppressed by fluvastatin (Figure 6C), and the co-treatment of both resulted in the increase of the sub-G1 population (Figure 6D). Mechanistically, these increases of the sub-G1 populations by the combined treatment were almost entirely blocked by the supplemented mevalonate in both SK-MEL-28 cells (Figure 6E) and A549 cells (Figure 6F), suggesting that the combinatorial apoptosis is dependent on the inhibition of the mevalonate pathway. On the other hand, the apoptosis observed in SK-MEL-28 cells and A549 cells was not negated by the addition of zVAD-fmk (Supplementary Figure 6A and 6B), the underlying mechanism of which could be caspase-independent unlike that in MDA-MB-231 cells (Figure 3A and 3B).

## DISCUSSION

**Figure 7:**
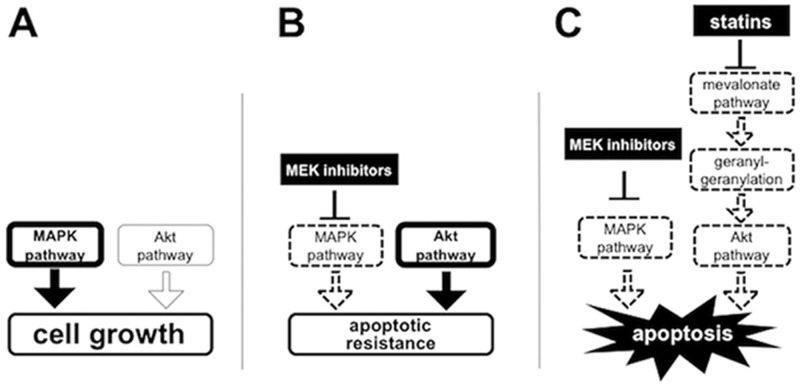
Schematic representation of the mechanisms of which statins overcome the apoptotic resistance to MEK inhibitors through the blockage of the mevalonate pathway Activated pathways are drawn with bold lines. Deactivated pathways are drawn with dotted lines. **(A)** MAPK pathway is dominantly activated to promote cell growth in untreated *KRAS* and/or *BRAF* mutated cancer cells. **(B)** MEK inhibition induces Akt activation and causes the apoptotic resistance to MEK inhibitors. **(C)** Blockage of the protein geranylgeranylation by statins suppresses Akt activation and thereby overcomes apoptotic resistance to MEK inhibitors.

We discovered two potent MEK inhibitors, trametinib and CH5126766, by the cell-based screening in collaboration with pharmaceutical companies [33]. Fortunately, it has been commonly recognized that trametinib is indispensable for treatment for *BRAF* mutated melanoma. Recently, trametinib was approved for non-small cell lung carcinoma (NSCLC) harboring *BRAF* mutation, which implies that MEK inhibitors could be applied to other cancers with activation of the MAPK pathway. However, the apoptotic resistance to MEK inhibitors is one of the most difficult problems in the expansion of the indication for MEK inhibitors. Considering that we originally discovered these MEK inhibitors by screening for RB-reactivating compounds [33], it is no wonder that they cannot induce apoptosis well because RB activation restrains the pro-apoptotic activity of E2F1 [34]. We here demonstrated that the blockade of the mevalonate pathway using statins overcomes the apoptotic resistance to MEK inhibitors with suppression of Akt activation (Figure 7). While there is growing evidence that several oncogenic signals confer resistance to MEK inhibitors, it is noteworthy that metabolic pathways, such as the mevalonate pathway, could be targetable to enhance the efficacy of MEK inhibitors.

The mevalonate pathway has been known to affect several malignant features of cancer, such as proliferation [35–37], cell survival [37, 38], cell invasion [39, 40], metastasis [39] and stemness [41]; however, limited information is available on the relationship between this metabolic pathway and drug resistance, especially regarding molecular-targeting agents. Recently, Chen *et al.* reported that atorvastatin overcame gefitinib resistance with the synergistic inhibition of MEK and PI3K/Akt pathways in *KRAS* mutant NSCLC cells [42], whose proof-of-concept was proven in a randomized study demonstrating the efficacy of gefitinib plus simvastatin in NSCLC patients [43]. This evidence raises expectation for the clinical trial of trametinib or CH5126766 plus statins in cancer patients with *BRAF* or *RAS* mutation. The doses of CH5126766 (20–40 nM), trametinib (5–40 nM) and fluvastatin (0.3 μM) used in our study are within the range of serum concentrations found in humans, supporting our proposal of this combination in the clinic.

The end products of the mevalonate pathway are generally classified into two metabolites: cholesterol and farnesyl pyrophosphate. It must be considered which one is cytoprotective and essential for drug resistance. For instance, in acute myeloid leukemia (AML) patients, cholesterol synthesis is abnormally upregulated during chemotherapy, and the inhibition of cholesterol synthesis led to sensitizing AML cells to therapeutics [44, 45]. Furthermore, in renal cell carcinoma, the efficacy of the mTOR inhibitor temsirolimus was associated with increases in serum cholesterol levels after treatment [46]. On the other hand, a recent report showed that trametinib and fluvastatin led to synergistic suppression of cancer-like phenotypes using a *Drosophila* transgenic lung cancer model [47], in which cholesterol is not synthesized because *Drosophila* use cholesterol from dietary sources, indicating that cholesterol biosynthesis is not critical for the efficacy of this combination. Indeed, we found that the addition of not cholesterol or FPP but GGPP almost fully cancelled the combined apoptosis of MEK inhibitor with statins (Figure 5E and 5F), indicating that the inhibition of protein geranylgeranylation is required for the apoptotic sensitization to MEK inhibition. More importantly, the pharmacological inhibition of geranylgeranylation using GGTI mimicked the effect of statins on the sensitivity to MEK inhibitors, suggesting that GGTI may be a good candidate for use with MEK inhibitors.

In our study, statins repressed the phosphorylation of Akt induced by MEK inhibition dependently on geranylgeranylation inhibition (Figure 5G). Protein geranylgeranylation leads to the activation of small GTPases, such as RhoA and Rac1, with binding to the cellular membrane. Indeed, RhoA promotes the binding of phosphorylated focal adhesion kinase to PI3K, resulting in Akt activation [48], while Rac1 forms the complexes with PI3K to stimulate PI3K-Akt signaling [49]. Thus, statins may suppress Akt activation through impeding the geranylgeranylation of RhoA or Rac1.

The dual inhibition of MEK and PI3K-Akt signaling is well known to lead to the upregulation of the apoptotic protein BIM to induce synergic apoptosis [30]. However, we did not observe significant upregulation of BIM by the co-treatment of MEK inhibitors with statins (Figure 3C and 3D). Recently, Berger *et al.* reported that the upregulation of BIM was not sufficient to induce RAF inhibitor-induced apoptosis and extrinsic apoptotic signaling, such as TRAIL stimulation, was required to trigger sufficient cell death in this context [31]. Indeed, in line with this evidence, we confirmed the upregulation of TRAIL induced by the co-treatment of CH5126766 with statins in MDA-MB-231 cells (Figure 3C and 3D), which at least partially contributed to the induction of apoptosis (Figure 3E and 3F). These results are unique in suggesting the possibility that cancer cells themselves secrete TRAIL after the deactivation of MEK and PI3K/Akt signaling. To our knowledge, there are few reports demonstrating cancer cell-mediated autocrine or paracrine secretion of TRAIL induced by the treatment of low molecule compounds: all-*trans* retinoic acid induced TRAIL in a paracrine mode in leukemia cells [50], while HDAC inhibitor MS275 induced endogenic TRAIL in several breast cancer cells [51]. However, we did not observe the upregulation of TRAIL in other cell lines, such as SK-MEL-28 and A549 cells (data not shown), which was consistent with our results showing that the combination of trametinib and fluvastatin induced apoptosis independently of caspase activation in these cell lines (Supplementary Figure 6). Thus, the causative mechanisms of TRAIL induction appear to be dependent on cellular context, and further studies will be required to precisely determine how and when TRAIL is induced in cancer cells.

In summary, we revealed that the deactivation of the mevalonate pathway evokes MEK inhibitor-mediated apoptosis with the suppression of Akt activation, suggesting that the metabolic dependency confers the apoptotic resistance to MEK inhibition. From the viewpoint of drug repositioning, fluvastatin, which was used in this study within a physiologically feasible concentration, could be expected as an apoptotic sensitizer of MEK inhibitor in clinical settings. While *in vivo* studies are needed to confirm the proof of this concept, the clinical study should be positively considered to examine whether the targeting the mevalonate pathway could enhance the efficacy of MEK inhibitors.

## MATERIALS AND METHODS

### Cell culture

Human breast cancer MDA-MB-231 cells and human melanoma SK-MEL-28 cells were obtained as NCI-60 from the National Cancer Institute Developmental Therapeutics Program (Bethesda, MD, USA). Human non-small cell lung cancer A549 cells were obtained from the American Type Culture Collection (Manassas, VA, USA). MDA-MB-231 cells were cultured in RPMI-1640 supplemented with 10% fetal bovine serum, 2 mM L-glutamine and antibiotics (50 U/ml penicillin and 100 μg/ml streptomycin). The other cell lines were cultured in DMEM supplemented with 10% fetal bovine serum, 4 mM L-glutamine and antibiotics (50 U/ml penicillin and 100 μg/ml streptomycin). Cells were incubated at 37°C in a humidified atmosphere of 5% CO_2_.

### Reagents

CH5126766 was kindly provided by Chugai Pharmaceutical Co., Ltd. (Tokyo, Japan). Trametinib was kindly provided by GlaxoSmithKline (Brentford, Middlesex, UK). Fluvastatin and simvastatin were purchased from LKT Labs (St Paul, MN, USA). zVAD-fmk was purchased from R&D Systems (Minneapolis, MN, USA). Zoledronate was purchased from Selleck Chemicals (Houston, TX, USA). Mevalonate, geranylgeranyl pyrophosphate, farnesyl pyrophosphate, cholesterol, GGTI-298 and FTI-277 were purchased from Sigma-Aldrich (St Louis, MO, USA). Mevalonate, FTI-277, cholesterol and zoledronate were dissolved in water. The other agents were dissolved in DMSO.

### Cell growth assay

The number of viable cells was assessed with a Cell Counting Kit-8 assay according to the manufacturer's instructions (Dojindo, Kumamoto, Japan). Cells were seeded at a density of 2,000 cells per well in 96-well plates and incubated for 24 h, and then treated with each agent for 72 h. After a further 4 h incubation with the kit reagent, the absorbance at 450 nm of the samples was measured using a multi-plate reader (DS Pharma Biomedical, Osaka, Japan).

### Colony formation assay

Cells were seeded at a density of 200 cells per well in 6-well plates. After incubating for 24 h, cells were treated with each agent for 48 or 72 h. The medium was then replaced with fresh medium. After further incubation of 14-17 days, the cells were fixed with 10% formalin and stained with 0.1% crystal violet.

### Analysis of apoptosis and cell cycle

Cells were seeded at a density of 1 × 10^5^ (MDA-MB-231 cells) or 5 × 10^4^ (SK-MEL-28 and A549 cells) cells per well in 6-well plates and incubated for 24 h. After treatment with the indicated agents for 48 or 72 h, cells were harvested by trypsinization. Following centrifugation, the cells were washed twice with PBS and stained in PBS containing 0.1% Triton X-100 and 25 μg/ml propidium iodide. Flow cytometry analysis was carried out with a FACSCalibur (Becton Dickinson, Franklin Lakes, NJ, USA). A total of 1 × 10^4^ cells was counted for each experiment. DNA fragmentation was quantified by the percentage of cells with hypodiploid DNA as the sub-G1 population. These data were analyzed with Cell Quest software and Modifit LT software (Becton Dickinson).

### Protein isolation and western blotting

Cells were harvested and lysed in the following lysis buffers: for unprocessed Rap1 detection, boiling hot lysis buffer containing 100 mM Tris-HCl, 1.1% SDS, 11% glycerol was used; for the other proteins, lysis buffer containing 50 mM Tris-HCl, 1% SDS, 1 mM DTT and 0.43 mM ABSF was used. After then, lysates of cells were sonicated and centrifuged at 20,400 *g* for 20 min at 4°C, and the supernatant was collected. Equal amounts of the protein extract were subjected to SDS-PAGE, and transferred to a PVDF membrane (Millipore, Bedford, MA, USA). The following were used as the primary antibodies: rabbit anti-Akt, rabbit anti-phospho-Akt (Ser473), rabbit anti-p44/42 MAPK (ERK1/2), rabbit anti-phospho-p44/42 MAPK (ERK1/2), rabbit anti-PARP (Cell signaling Technology, Beverly, MA, USA), mouse anti-β-actin (Sigma-Aldrich), rabbit anti-BIM (Abcam, Cambridge, UK), mouse anti-TRAIL (Santa Cruz Biotechnology, Santa Cruz, CA, USA), rabbit anti-HMGCR (Abcam) and mouse anti-Rap1 (Santa Cruz Biotechnology). The signals were detected with a Chemi-Lumi One L (Nacalai Tesque) or an Immobilon™ Western Chemiluminescent HRP Substrate (Millipore).

### Small interfering RNA transfection

Small interfering RNAs (siRNA) were obtained from Invitrogen (Carlsbad, CA, USA). The following siRNAs were used: siTRAIL #1 (10620318-241607 D09; Stealth RNAi™ siRNA), ACCUGCGUGCUGAUCGUGAUCUUCA; siTRAIL #2 (10620318-242264 E05; Stealth RNAi™ siRNA), AAUCAUCAAGGAGUGGGCAUUCAUU; and a negative control (12935-112; Stealth RNAi™ siRNA negative control Med GC duplex #2). Cells were seeded at a density of 5×10^4^ cells per well in 6-well plates. After incubating for 24 h, cells were transfected using lipofectamine RNAiMAX (Invitrogen) according to the manufacturer's instructions. Twenty-four hours after the transfection, the cells were treated with agents for 72 h and then harvested.

### Cholesterol assay

Intracellular cholesterol concentrations were assessed with a Total Cholesterol Assay Kit (Cell Biolabs, San Diego, CA, USA) according to the manufacturer's instructions. Briefly, cells were seeded into 10 cm dishes and harvested by trypsinization. We washed the cells with PBS, then extracted lipids with the kit lysis buffer and homogenized by sonication. The samples were centrifuged at 15,000 *g* for 5 min at 4°C to obtain the organic phase. The obtained extracts were air dried at 50°C for 15 min and vacuumed for 30 min, and then the dried lipids were resuspended with the kit diluent buffer and sonicated. After a further 45 min incubation with the kit reagent, the absorbance at 550 nm of the samples was measured using a multi-plate reader (DS Pharma Biomedical).

### Statistical analysis

All results are presented as mean ± standard deviation (SD). Statistical difference of means between two groups was assessed using an unpaired Student's *t* test, and P-values less than 0.05 were considered significant.

## SUPPLEMENTARY MATERIALS FIGURES AND TABLES


