# Supplementary material for: Blockage of the mevalonate pathway overcomes the apoptotic resistance to MEK inhibitors with suppressing the activation of Akt in cancer cells
Source: Oncotarget. 2018 Apr 13;9(28):19597–612. doi: 10.18632/oncotarget.24696 (PMC5929411; doi:10.18632/oncotarget.24696)
Supplement: Supplementary file 1 [file oncotarget-09-19597-s001.pdf]

Blockage of the mevalonate pathway overcomes the apoptotic resistance to MEK inhibitors with suppressing the activation of Akt in cancer cells

SUPPLEMENTARY MATERIALS

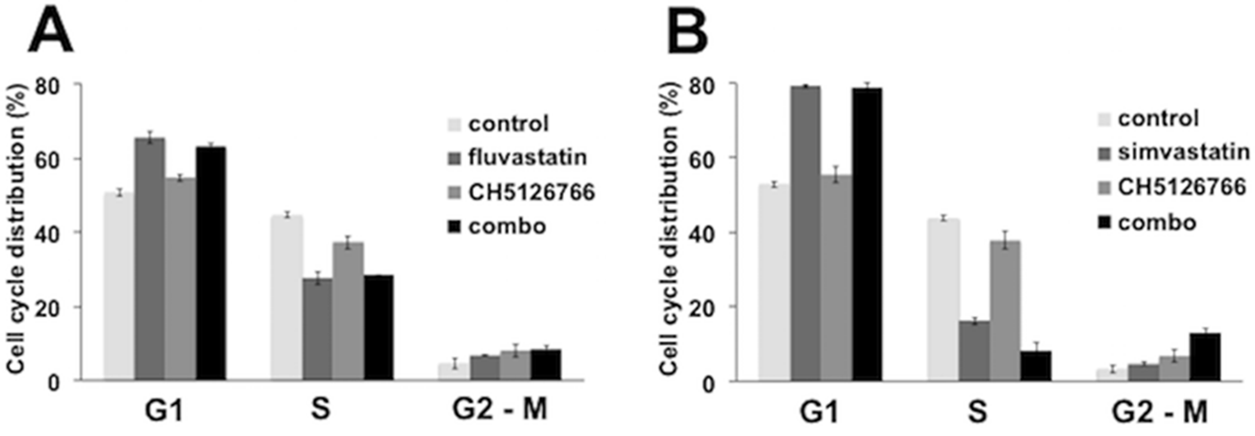

Supplementary Figure 1: Statins with or without CH5126766 induce G1 arrest in MDA-MB-231 cells. **(A)** Cell cycle analysis after the combined treatment of CH5126766 with fluvastatin. Cells were treated with CH5126766 (40 nM) and/or fluvastatin (0.3 μM) for 72 h, and the DNA contents of the cells were analyzed by flow cytometer. The percentages in each phase of the cell cycle are shown. Columns, means of triplicate data; bars, standard deviation (SD). **(B)** Cell cycle analysis after the combined treatment of CH5126766 with simvastatin. Cells were treated with CH5126766 (20 nM) and/or simvastatin (0.3 μM) for 72 h, and the DNA contents of the cells were analyzed by flow cytometer. The percentages in each phase of the cell cycles are shown. Columns, means of triplicate data; bars, SD.

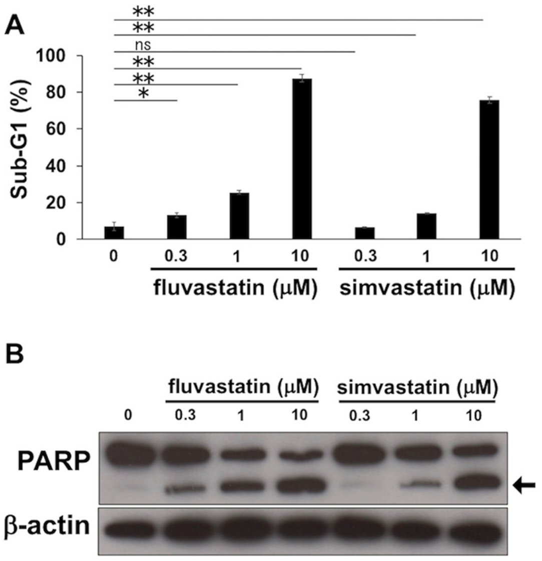

Supplementary Figure 2: Statins induce dose dependent increases in apoptosis in MDA-MB-231 cells. **(A)** The sub-G1 populations after the treatment of fluvastatin or simvastatin. Cells were treated with fluvastatin or simvastatin at the indicated concentrations for 72 h. DNA contents of the cells were analyzed by flow cytometer. Columns, means of triplicate data; bars, SD; **, $P < 0.01$; *, $P < 0.05$; ns, no significant difference. **(B)** The cleavage of PARP after the treatment of fluvastatin or simvastatin. Cells were treated with fluvastatin or simvastatin at the indicated concentrations for 48 h, and cleaved PARP was analyzed by Western blotting. The arrow indicates the cleaved form of PARP. β-Actin was used as a loading control.

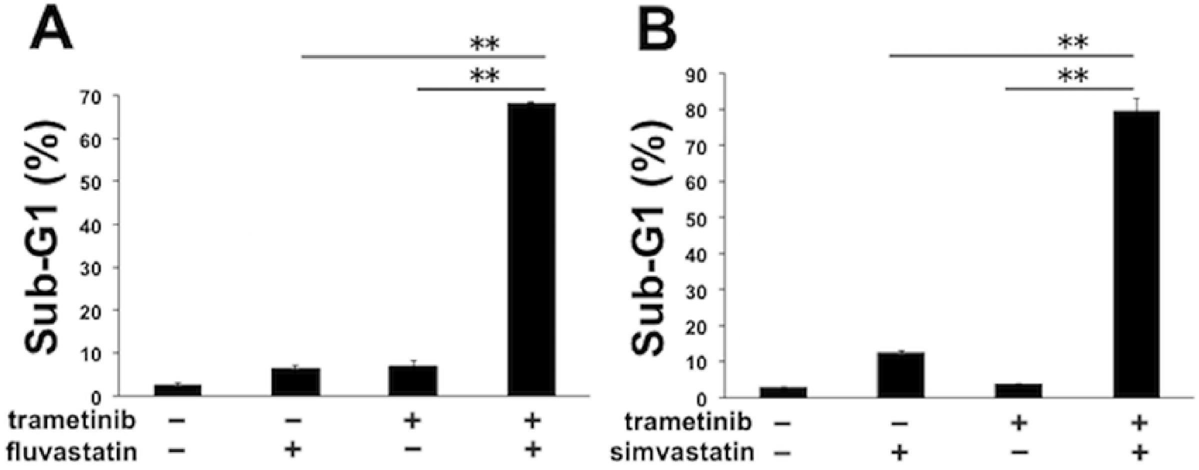

Supplementary Figure 3: The combined treatment of trametinib with statins induces apoptosis in MDA-MB-231 cells. **(A)** The sub-G1 populations after the combined treatment of trametinib with fluvastatin. Cells were treated with trametinib (40 nM) and/or fluvastatin (0.3 μM) for 72 h. DNA contents of the cells were analyzed by flow cytometer. Columns, means of triplicate data; bars, SD; **, $P < 0.01$. **(B)** The sub-G1 populations after the combined treatment of trametinib with simvastatin. Cells were treated with trametinib (20 nM) and/or simvastatin (0.3 μM) for 72 h. DNA contents of the cells were analyzed by flow cytometer. Columns, means of triplicate data; bars, SD; **, $P < 0.01$.

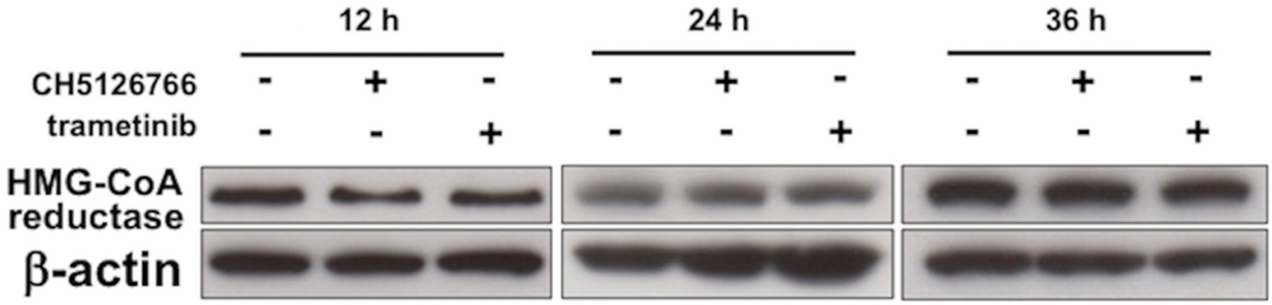

Supplementary Figure 4: MEK inhibitors do not alter the expression levels of HMG-CoA reductase in MDA-MB-231 cells. Cells were treated with CH5126766 (40 nM) or trametinib (40 nM) for the indicated times, and HMG-CoA reductase was analyzed by Western blotting. β-Actin was used as a loading control.

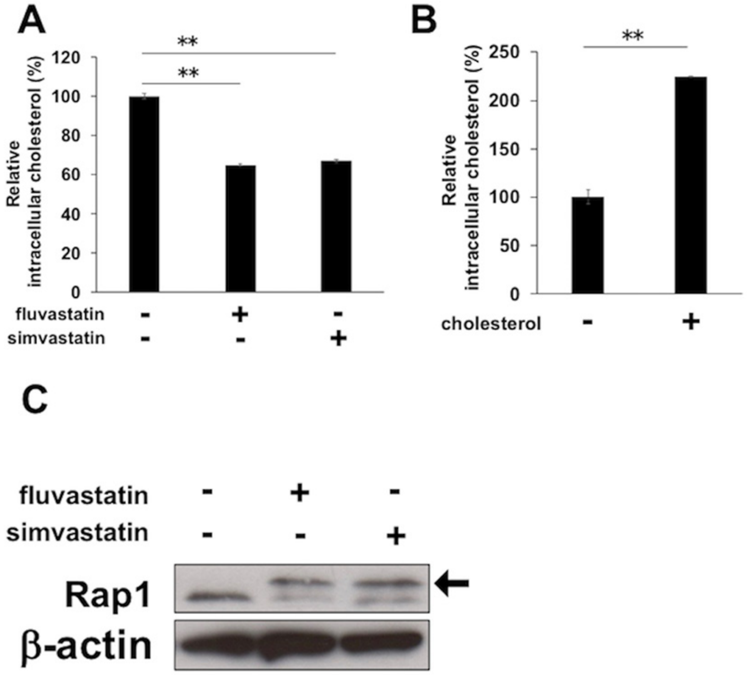

Supplementary Figure 5: Statins block the mevalonate pathway, including both of cholesterol biosyntheses and protein geranylgeranylation. (A) Intracellular cholesterol suppression by statins. MDA-MB-231 cells were treated with fluvastatin (0.3 μM) or simvastatin (0.3 μM) for 24 h, and the intracellular cholesterol levels were measured with a Total Cholesterol Assay kit. Obtained data were normalized to cell numbers. Columns, means of triplicate data; bars, standard deviation (SD); **, $P < 0.01$. **(B)** Intracellular cholesterol increase after the supplementation of cholesterol. MDA-MB-231 cells were incubated with cholesterol (50 μM) for 24 h, and the intracellular cholesterol levels were measured with a Total Cholesterol Assay kit. Obtained data were normalized to cell numbers. Columns, means of triplicate data; bars, standard deviation (SD); **, $P < 0.01$. **(C)** Processing of Rap1 by statins. MDA-MB-231 cells were treated with fluvastatin (0.3 μM) or simvastatin (0.3 μM) for 12 h, and Rap1 was analyzed by Western blotting. The arrow indicates the unprocessed form of Rap1. β-Actin was used as a loading control.

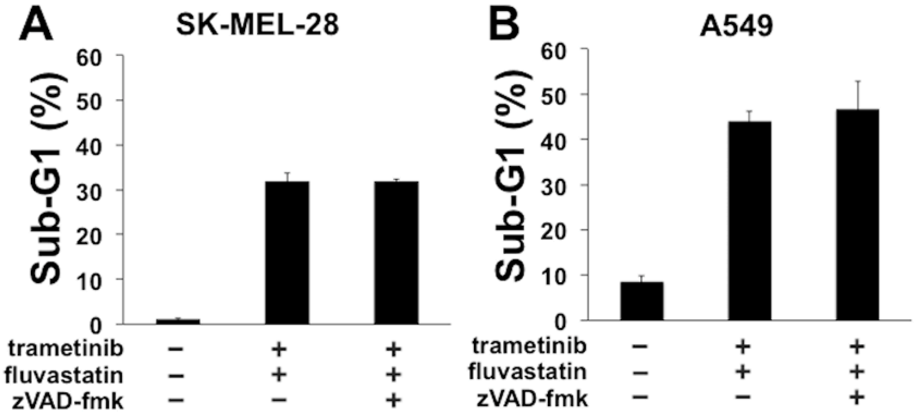

Supplementary Figure 6: Apoptosis induced by trametinib with fluvastatin is independent of caspase in SK-MEL-28 and A549 cells. (A) The sub-G1 populations after the combined treatment of trametinib with fluvastatin in the absence or presence of the pan caspase inhibitor zVAD-fmk in SK-MEL-28 cells. SK-MEL-28 cells were treated with trametinib (5 nM) and fluvastatin (1 μM) for 72 h with or without zVAD-fmk (20 μM). Columns, means of triplicate data; bars, SD. **(B)** The sub-G1 populations after the combined treatment of trametinib with fluvastatin in the absence or presence of the pan caspase inhibitor zVAD-fmk in A549 cells. A549 cells were treated with trametinib (40 nM) and fluvastatin (2 μM) for 72 h with or without zVAD-fmk (20 μM). DNA contents of the cells were analyzed by flow cytometer. Columns, means of triplicate data; bars, SD.
